# The global burden of sepsis: barriers and potential solutions

**DOI:** 10.1186/s13054-018-2157-z

**Published:** 2018-09-23

**Authors:** Kristina E. Rudd, Niranjan Kissoon, Direk Limmathurotsakul, Sotharith Bory, Birungi Mutahunga, Christopher W. Seymour, Derek C. Angus, T. Eoin West

**Affiliations:** 1 International Respiratory and Severe Illness Center, University of Washington, Seattle, WA USA; 2 Division of Pulmonary, Critical Care, and Sleep Medicine, Department of Medicine, University of Washington, Seattle, WA USA; 3 Department of Critical Care Medicine, University of Pittsburgh School of Medicine, 3550 Terrace St., Scaife Hall, #639, Pittsburgh, PA USA; 4 Division of Critical Care, Department of Pediatrics, University of British Columbia, British Columbia Children’s Hospital, Vancouver, BC Canada; 5 Mahidol Oxford Tropical Medicine Research Unit, Mahidol University, Bangkok, Thailand; 6 Centre for Tropical Medicine and Global Health, Nuffield Department of Medicine, University of Oxford, Oxford, UK; 7 Division of Infectious Diseases, Department of Medicine, Calmette Hospital, Phnom Penh, Cambodia; 8 Bwindi Community Hospital, Kanungu, Uganda; 9 Departments of Critical Care and Emergency Medicine, University of Pittsburgh School of Medicine, Pittsburgh, PA USA; 10 Department of Critical Care Medicine, University of Pittsburgh School of Medicine and UPMC Health System, Pittsburgh, PA USA; 11 Department of Global Health, University of Washington, Seattle, WA USA

**Keywords:** Health resources, Poverty, Public health, Sepsis, Low-income countries

## Abstract

Sepsis is a major contributor to the global burden of disease. The majority of sepsis cases and deaths are estimated to occur in low and middle-income countries. Barriers to reducing the global burden of sepsis include difficulty quantifying attributable morbidity and mortality, low awareness, poverty and health inequity, and under-resourced and low-resilience public health and acute health care delivery systems. Important differences in the populations at risk, infecting pathogens, and clinical capacity to manage sepsis in high and low-resource settings necessitate context-specific approaches to this significant problem. We review these challenges and propose strategies to overcome them. These strategies include strengthening health systems, accurately identifying and quantifying sepsis cases, conducting inclusive research, establishing data-driven and context-specific management guidelines, promoting creative clinical interventions, and advocacy.

## Background

Sepsis, a syndrome of dysregulated host response to infection leading to life-threatening organ dysfunction, is a substantial health burden worldwide. More than 19 million sepsis (formerly severe sepsis) cases and 5 million sepsis-related deaths are estimated to occur annually—the majority in low and middle-income countries (LMICs) [1]. There is tremendous range in the capacity of public health and acute health care delivery systems across and within LMICs. Here, we target the challenge of sepsis in low-resource settings. These low-resource settings are more prevalent in, although not exclusively confined to, LMICs. There is heterogeneity among these low-resource areas, including important regional, political, and economic differences. Still, these regions are frequently plagued by low-resilience health systems and are home to people with limited access to health care.

While there are some published population-level estimates of sepsis epidemiology in high-income countries (HICs), the methods and results of these studies are highly variable [2–4]. In contrast, the burden in LMICs remains decidedly understudied [1, 5]. Also, while outcomes of septic patients in HICs have improved in recent decades [2, 6], there is little evidence to support a similar trend in LMICs. Many barriers exist to reducing the global burden of sepsis, particularly in low-resource settings. Here, we review these hurdles and outline an integrated strategy to overcome them. This review primarily targets public health policymakers and funders as well as critical care and acute health care system leaders working in low-resource settings. While extensive examination of every facet of sepsis prevention, identification, and management is prohibitive, selected elements represent themes in the scientific literature and included references serve as examples of these themes rather than statements meant to represent all low-resource settings or the entire body of available evidence.

## Quantifying the global burden of sepsis

### Challenges in definitions

Early, accurate identification of patients with sepsis is critical to improving outcomes through better targeted medical management [7], yet remains challenging. There is no single “gold standard” diagnostic test for sepsis, and case definitions vary widely. Use of nonspecific terminology such as “septicemia” remains pervasive. A consensus definition of adult sepsis, originally proposed in 1991, was most recently revised in 2016 (Sepsis-3) [8, 9]. Sepsis-3 defined sepsis as a syndrome of “life-threatening organ dysfunction caused by a dysregulated host response to infection” [9], and the authors proposed that organ dysfunction be identified by a change in organ failure score relative to baseline. The analyses underpinning these clinical criteria were based on data exclusively drawn from the United States and Germany [10]. The validity of one Sepsis-3 clinical prompt, the Quick Sequential (Sepsis-related) Organ Failure Assessment (qSOFA) score, has now been retrospectively evaluated in multiple datasets from LMICs [11]. However, neither the qSOFA score nor other Sepsis-3 criteria have been tested prospectively in low-resource settings. Until these clinical criteria are more fully validated in more diverse populations, their utility elsewhere in the world remains uncertain. Additionally, the Sepsis-3 Task Force explicitly did not examine definitions of infection and did not specify which infections, when leading to life-threatening organ dysfunction, should be considered as causes of sepsis [9]. There is some disagreement within sepsis and infectious disease communities regarding this issue; for example, some authors consider malaria to be a potential cause of sepsis, whereas others do not. This disagreement has important implications for comparability of patient populations in research studies and for the clinical application of sepsis treatment guidelines.

The definition of pediatric sepsis is also challenging. Last revised in 2005 and based heavily on the 1991 adult sepsis consensus definition, the pediatric definition offers age-appropriate values for the systemic inflammatory response syndrome (SIRS) criteria [12]. Combined with suspected infection, the presence of these modified SIRS criteria is used to identify pediatric patients with sepsis. This definition has not been revisited in over a decade and has posed problems in implementation, especially in low-resource settings, due to its low specificity and requirement for a leukocyte count [13].

### Heterogeneous clinical use of definitions

While the adult and pediatric consensus sepsis definitions are endorsed by multiple professional societies, clinicians show high levels of disagreement regarding the application of the definitions to clinical cases [14]. Additionally, the sepsis definitions are currently operationalized through the use of various clinical scoring systems (such as the qSOFA score, SOFA score, or SIRS criteria), some of which may be impractical to use at the bedside in low-resource settings. Moreover, the burden of sepsis is very difficult to quantify when clinicians do not uniformly document it in their practice. Often patients are classified based on the primary source of infection, such as pneumonia or meningitis, but are not explicitly deemed to have sepsis [15]. Accurate assessment of sepsis epidemiology is most feasible on a broad scale through the use of International Classification of Diseases (ICD) coding in administrative data or vital records, but this is impossible without more uniform coding practices worldwide.

### Initiatives to quantify the burden

As a result of these issues related to sepsis definition and documentation, most estimates of the global burden of sepsis are likely to be inaccurate. Further, it is likely that many sepsis deaths occur at home, particularly in low-resource settings, and thus are less likely to be identified and documented as sepsis related. Two of the most comprehensive and rigorous efforts to document the worldwide burden of disease, the Global Burden of Disease study by the Institute for Health Metrics and Evaluation (IHME) [16] and the World Health Organization’s (WHO) World Health Statistics [17], have not previously quantified the burden of sepsis beyond neonatal or maternal sepsis. Encouragingly, the IHME has recently indicated that it will produce estimates for the burden of sepsis for the first time, and the World Health Assembly of the WHO passed a resolution on Improving the Prevention, Diagnosis, and Management of Sepsis in May 2017 [18].

## Sepsis highlights health system challenges

**Fig. 1:**
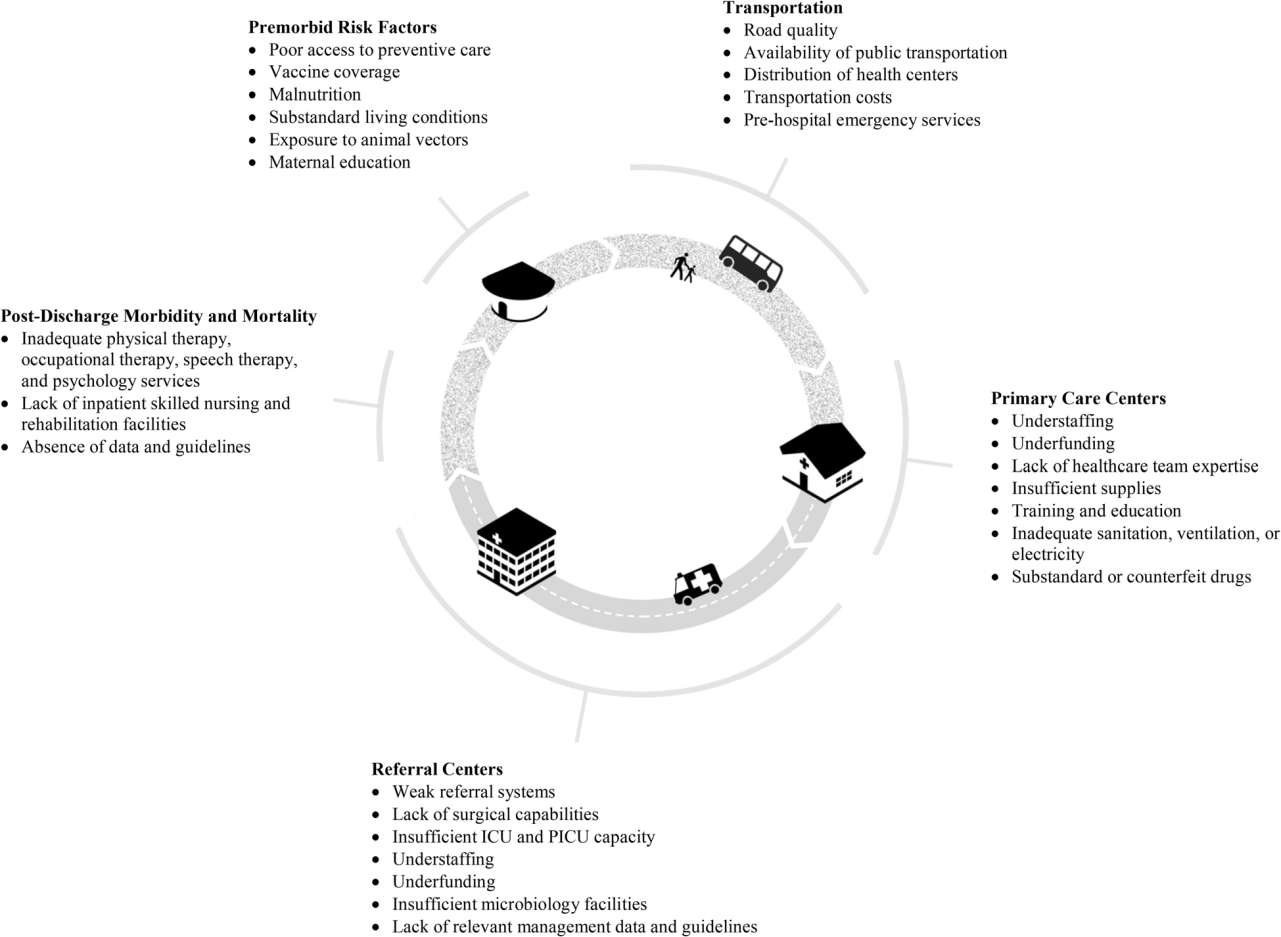
Burden of sepsis highlights public health and acute health care delivery system challenges. ICU intensive care unit, PICU pediatric intensive care unit

**Fig. 2:**
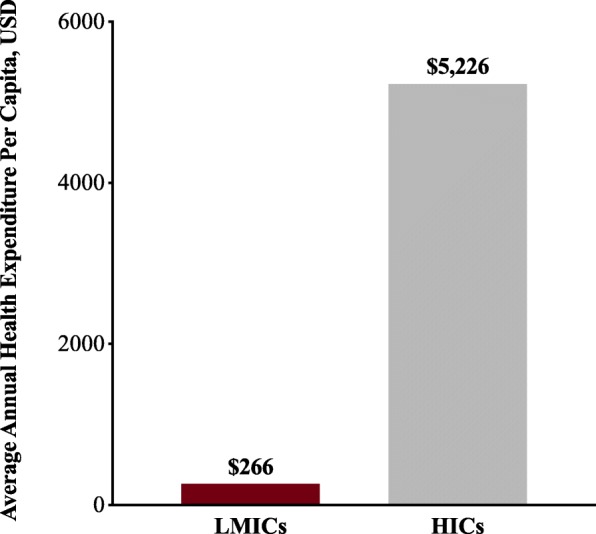
Annual health expenditure per capita in high-income countries (HICs) versus low or middle-income countries (LMICs). The 2014 health expenditure per capita from World Bank’s DataBank Health, Nutrition, and Population Statistics database [23]

In low-resource settings, poverty, political corruption, health inequity, and under-resourced and low-resilience public health and acute health care delivery systems are fundamental contributors to the burden of sepsis (Fig. 1). For example, poverty-associated conditions such as poor preventive health care, limited vaccine coverage, malnutrition, substandard living conditions such as indoor air pollution, bed sharing, and inadequate ventilation and sanitation, and exposure to environmental and animal vectors increase risk for acute infection [19–22]. These factors are further exacerbated by disparate funding of health systems (Fig. 2), delays in identifying and reaching appropriate care, and inadequate systems to prevent health care-associated infections [23, 24]. Addressing these issues, which are broadly relevant to health, is likely the greatest but perhaps the most challenging approach to reduce the global burden of sepsis. Moreover, given the significant variability within and across LMICs, it is critical to recognize that health system challenges must be evaluated at the community level, and the issues outlined in the following cannot be uniformly generalized to all areas of all LMICs.

### Human resources

Health care facilities frequently lack the personnel to care for patients with sepsis. Indeed, 83 countries—all of which are in Latin America, Africa, and Asia—do not meet the WHO standards for minimum health care worker-to-population ratios [25]. Inadequate staffing of health care centers and gaps in providers’ knowledge can impede rapid identification and treatment of patients with sepsis [26]. These knowledge gaps may be greater for pediatric sepsis care, as many areas lack pediatricians, and providers who do not routinely care for children may be uncomfortable with the nuances of pediatric resuscitation.

### Facilities

Many countries do not have sufficient health care facilities to care for the population they serve, with overcrowded medical wards or long distances between patients’ homes and the nearest health center. The available facilities are sometimes in poor condition, with inadequate sanitation, ventilation, electrical supply, or lighting to allow for safe patient care [27]. This can impede appropriate care and contribute to the spread of health care-acquired infections.

### Surgical capacity

Capacity for timely surgical intervention to eradicate infection may be limited due to a combination of inadequate facilities, essential medications, supplies, and human resources (including surgeons and anesthesiologists) [28]. At least 4.8 billion people (67% of the world’s population) do not have access to surgery—including more than 95% of the population in South Asia and central, eastern, and western sub-Saharan Africa [28]. Based on current surgical rates and rates of growth in surgical capacity, 73% of the world’s population will still be living in countries below the minimal recommended surgical rate in 2035 [29]. Pediatric surgical capacity is particularly problematic [30].

### Critical care capacity

Critical care capacity is suboptimal worldwide, with inadequate high acuity and intensive care unit (ICU) facilities, providers, and supplies [31]. ICUs are often so chronically overcrowded that they are unable to accommodate critically ill patients with sepsis or septic shock, resulting in patients requiring vasopressors or mechanical ventilation being managed outside of the ICU—where the degree of monitoring is highly variable, if not absent. In some countries, ICU care is only accessible to the wealthy. Even where high-quality critical care facilities are available, allocation of scarce resources may not be applied in a transparent, consistent, equitable, or accountable manner [32].

### Supplies and diagnostic capacity

Many hospitals lack the requisite resources to implement current sepsis management guidelines [33]. Lack of intravenous (IV) fluids, supplemental oxygen, simple positive pressure airway systems, and basic monitoring equipment such as pulse oximeters is common in low-resource settings [34]. The placement of central venous catheters in a safe and sterile manner is often difficult, especially in children.

This unequal global distribution of medical resources may also limit resuscitation and organ support provided to septic patients in low-resource settings. For example, clinicians may avoid interventions deemed at higher risk, such as IV fluid resuscitation in a patient with respiratory insufficiency in a setting without available oxygen support or mechanical ventilation, for fear of doing harm by worsening pulmonary edema and respiratory failure [35]. Many guidelines and research studies have focused on bundled care [36, 37], which can be challenging for clinicians who are unable to administer all features of a bundle. It is often difficult or impossible to discern which features of a bundled management approach are most effective—and cost-effective—in reducing sepsis morbidity and mortality [38, 39].

There is a paucity of adequate microbiological diagnostic capacity in many settings, limiting clinicians’ ability to tailor antimicrobial therapy to local pathogen and resistance profiles or individual patients’ pathogen sensitivity and resistance patterns, as is recommended in many sepsis management guidelines [40]. Diagnostic capacity extends beyond just physical components of microbiology laboratories such as reliable access to consumable supplies, and includes staffing with adequate numbers of appropriately trained professionals such as phlebotomists and laboratory technicians, as well as necessary oversight and enforcement of good clinical laboratory practice [41].

### Inadequate, inferior, and misused antimicrobials

In many low-resource settings, critical antimicrobials may not be available due to drug shortages, cost, or local import and regulation systems. Improving timely access to appropriate, effective, and affordable antimicrobials is of utmost importance. Counterfeit or substandard medications are a major problem, particularly where regulatory agencies are absent or ineffective, impacting millions of people annually [42, 43]. Consequently, use of these medications can result in inadequate treatment of infection, increasing morbidity and mortality. Other potential consequences include exacerbation of antimicrobial resistance (AMR) or adverse effects due to exposure to contaminants or unknown ingredients.

Overuse and abuse of antimicrobials is a major challenge in both low and high-resource settings. In many countries, antimicrobials are available without prescription in community pharmacies, potentially delaying care and fueling antibiotic resistance. However, these risks must be balanced against the potential benefits of increased access to medications in settings with few trained health care workers or limited access to appropriate medical care. Improper physician prescribing practices, fueled by unregulated pharmaceutical company marketing to prescribers, can also contribute to antimicrobial misuse [44, 45]. Poor antibiotic stewardship due to lack of access to adequate microbiology and diagnostic tests, and inadequate infectious disease and microbiology subspecialty expertise, can contribute to the increasing burden of AMR infections in LMICs [46–48].

AMR may be a major driver of the global burden of both community and hospital-acquired sepsis. More than 700,000 deaths per year may be attributable to AMR infections globally, and this number could reach 10 million by 2050 if the current trend is not addressed [49]. Additionally, while patients in HICs with drug-resistant infections usually have access to alternative (and often more expensive) antibiotics, many in LMICs do not. Unfortunately, AMR data are not available for many LMICs (e.g., one recent review failed to identify any AMR data from 43% of African countries), and the quality of microbiological data is highly variable [50]. In areas of the world for which data are available, the level of resistance is frequently alarming [50–52]. The prevalence of AMR organisms has important implications for management of sepsis, particularly in areas without access to robust clinical microbiology facilities.

### Addressing acute complications of sepsis

Many health systems are unable to adequately address acute complications of sepsis. For example, venous thromboembolism prophylaxis is not appropriately employed in much of the world, and may be unavailable in low-resource settings [53]. Many hospitals in LMICs are unable to provide acute renal replacement therapies for the upwards of 30% of sepsis patients who develop acute kidney injury [54, 55]. This lack of resources to manage life-threatening acute complications of sepsis almost certainly substantially increases sepsis-associated mortality in low-resource settings, even when the underlying infection may be treatable. The risk of developing a secondary health care-associated infection may be up to 20 times higher in LMICs than in HICs [56], placing patients at risk for developing a second sepsis episode due to an antimicrobial resistant organism and increasing the burden on the health system.

### Postdischarge morbidity and mortality

Long-term morbidity and mortality must be considered when evaluating the burden of sepsis globally. For example, one in every five pediatric sepsis survivors has a new functional disability [57]. Many health systems are unable to address short and long-term physical and psychological debility related to sepsis. Physical therapy, occupational therapy, rehabilitation medicine, and nursing care facilities are absent in many low-resource settings, impacting the functional outcomes of sepsis survivors and forming a significant barrier to reducing the burden of disability due to sepsis worldwide.

Among children, postdischarge mortality in LMICs is similar to, if not even higher than, in-hospital mortality [58]. This postdischarge mortality burden is borne by patients with identifiable risk factors, and these deaths occur early during the postdischarge period. While these observations suggest there may be early opportunities to decrease the burden and mortality [59], these issues remain challenging even in highly resourced settings, and are vastly understudied or addressed in low-resource settings.

## Sepsis care in high-resource settings may not be effective in low-resource settings

In the absence of robust data to guide optimal sepsis care in low-resource settings, caution must be taken in extrapolating from data generated in high-resource settings. Several characteristics of sepsis in low-resource settings, such as differences in populations at risk, infecting pathogens, and clinical circumstances, suggest that sepsis care in high-resource settings may not be effective in low-resource settings (Table 1).

**Table 1:** Reasons why sepsis care in high-resource settings may not be comparably effective in low-resource settings

Populations at risk • Poor access to preventive health care • Unidentified and inadequately controlled comorbidities • HIV • Malnutrition • Genetic features of immune response	
Infecting pathogens • Parasitic, viral, and mycobacterial infections • Antimicrobial resistance • Health care-associated infection	
Clinical circumstances • Limited critical care capacity • Inadequate human resources • Lack of large, high-quality, prospective sepsis studies in LMICs • Insufficient implementation science research	

*HIV* human immunodeficiency virus, *LMIC* low and middle-income country

### Differences in hosts

Key patient characteristics—comorbidities such as human immunodeficiency virus (HIV) or malnutrition, variability in environmental factors, or genetic features of host response—modify how sepsis presents in some LMIC settings. The high prevalence of HIV among patients with sepsis in some parts of the world dramatically changes the microbiologic profile and thus the antimicrobial strategy. Malnutrition significantly alters the presentation and management of sepsis, particularly among pediatric patients [60]. Fluid resuscitation and antimicrobial strategies must take this important comorbidity into account.

### Differences in pathogens

While bacterial infections account for a large majority of sepsis in HICs [61], sepsis in LMICs is characterized by different types of bacteria, including mycobacteria, and much higher proportions of nonbacterial organisms such as parasites and viruses [62]. *Escherichia coli*, *Enterococcus faecalis*, and *Staphylococcus aureus* are commonly isolated causes of sepsis in the United States [61, 63], and *E. coli* and *S. aureus* have also been found to be common causes of bacteremia in Thailand [64]. However, invasive nontyphoidal salmonella is an important cause of bacteremia in Africa; a meta-analysis of community-acquired bloodstream infections in Africa found that *Salmonella enterica* was the most prevalent isolate overall and in adults, and *Streptococcus pneumonia* was the most common isolate in children [65, 66]. Melioidosis (infection with *Burkholderia pseudomallei*) is the second most common cause of bacteremia in northeast Thailand [67]. There is a very high prevalence of tuberculous mycobacteremia among septic patients in Uganda and Malawi [68, 69].

Many patients in LMICs develop sepsis due to nonbacterial infections. For example, rickettsiosis is commonly found in sepsis patients throughout Southeast Asia [70]. Some studies have reported decreased sepsis mortality by empirically treating malaria [71]. Malaria and other acute parasitic infections, while often presenting in comparable fashion to bacterial sepsis, merit specific treatment [72]. Dengue virus is another common cause of sepsis in LMICs. This infection, characterized by severe thrombocytopenia and sometimes associated with profound extravasation of intravascular fluid, may require unique resuscitation strategies.

### Differences in clinical environments

The very different clinical environments in low-resource settings may also contribute to the nonapplicability of standard management strategies in high-resource settings. RCTs conducted in critically ill patients in low-resource settings reinforce this concern. A single-center RCT of adult patients with severe sepsis in Zambia was stopped early due to higher mortality among patients with hypoxemic respiratory failure in the IV fluid intervention arm [38]. An RCT of early versus late enteral feeding among cerebral malaria patients in Bangladesh found increased incidence of aspiration pneumonia in the group receiving early feeding [73]. Limited nursing personnel, ICU capacity, and availability of mechanical ventilation may have contributed to these adverse outcomes in the intervention groups.

The Fluid Expansion as Supportive Therapy trial demonstrated the dangers of aggressive IV fluid resuscitation in a population of East African febrile children who had some features of impaired circulation [74]. Additionally, a protocol for early resuscitation with IV fluids and vasopressors among adults (most of whom were HIV-positive) with sepsis and hypotension in Zambia resulted in higher in-hospital mortality compared with usual care [75]. With this evidence of harm in both adult and pediatric patients with sepsis and septic shock in Africa, and the absence of high-quality clinical trials in similar settings demonstrating any benefit of aggressive fluid resuscitation in sepsis, there is essentially no context-specific evidence supporting recommendations for fluid resuscitation in sepsis. Despite this lack of evidence, aggressive fluid resuscitation is advocated in some international and local LMIC sepsis management guidelines [37, 76]. These examples underscore the need for caution in the implementation of best practices from high-resource settings to other settings and populations in which they have not been validated.

## Practical steps to reduce the global burden of sepsis

### Accurately identify and quantify sepsis cases

To reduce the global burden of sepsis, several steps are necessary (Table 2). First, international adoption of a single clear, cohesive definition of sepsis is critical. Importantly, implementation of this definition and its associated clinical criteria must be practical in low-resource settings and needs to take into account the need for syndromic diagnoses in some areas of the world. Countries and health systems must make efforts to improve and standardize ICD coding practices in administrative data and vital records, and to educate clinicians on appropriate coding for sepsis. This is challenging in all settings, but is particularly so in hospitals with limited medical records capacity, such as those without access to electronic medical records systems and with very little staffing (or medical coding expertise). Comprehensive, rigorous data collection (particularly in low-resource settings, where there is the largest gap in reporting) is a necessary component of any efforts to improve sepsis care. Better data collection will lead to more sophisticated assessment of the impact of interventions and can help to guide appropriate public health planning and investments in health infrastructure. In addition to sepsis-specific data collection, it is important to strengthen national and international data collection systems to include registries of patients with suspected infection, a subset of whom will have sepsis.

**Table 2:** Essential steps to reduce the burden of sepsis in low-resource settings

Strengthen public health and acute health care delivery systems • Directly confront poverty and wealth inequalities • Prevent the spread and acquisition of infectious diseases • Accessible high-quality primary health care • Functional public transportation and prehospital emergency medical services • Strong, staffed, and well-supplied referral centers accessible to all • Increased critical care capacity, both within and outside of ICUs	
Accurately identify and quantify sepsis cases • Nuanced operationalization of sepsis definitions • Comprehensive, rigorous, population-level, sepsis-specific data collection in LMICs	
Conduct inclusive research • Increased partnership of adult and pediatric research communities • Integrate sepsis research with disease-specific research and clinical communities • Clinical trials in austere environments • Implementation science research • Open data access	
Establish data-driven and context-specific management guidelines • Partner with clinicians, public health professionals, and researchers to develop appropriate guidelines • Validate recommendations in varied populations • Focus on high-yield, cost-effective, and readily available interventions • Balance disease-specific recommendations with general approaches	
Promote creative interventions • Timing and route of antimicrobials • Diagnostics • Organ support	
Advocacy • Support international sepsis initiatives • Recognize sepsis as a major public health threat	

*ICU* intensive care unit, *LMIC* low and middle-income country

### Strengthen public health and acute health care delivery systems

Population-level sepsis incidence and outcomes are heavily driven by the strengths (and weaknesses) of public health and acute health care delivery systems. The most critical interventions to reduce the global burden of sepsis will ultimately improve public health overall and create health benefits far beyond sepsis. The fundamental driver of suboptimal capacity to prevent, identify, and treat sepsis in low-resource settings is health inequity, largely driven by economic disparity. Any public health initiative to reduce the burden of sepsis must consider how to reduce health disparities and address issues of poverty. This includes integrating sepsis programs of multilateral organizations such as the WHO into broader initiatives targeting health effects of poverty more generally. In order to strengthen public health and acute health care delivery systems in the areas with the greatest burden of sepsis, LMIC health systems must have more funding—this requires changes in global and national-level economic policies.

Efforts to prevent the spread and acquisition of infectious diseases are critical, such as those focused on vaccination, nutrition, maternal education, healthy living environments (bed nets, clean cook-stove options, and sanitation), and safe drinking water. There must be focus on the prevention of hospital-acquired infection as well. Once patients develop an acute infection, reliable and safe transportation to an appropriate health facility must be available. Prehospital care systems need to be established where feasible and strengthened where they do exist. Prehospital emergency care providers should be trained in the identification and early management of patients with sepsis, an intervention that has been associated with reduced mortality [77].

Once patients with sepsis reach health care facilities, they must have access to the basic personnel and material resources necessary for treatment, including surgical expertise. This will require sepsis-specific education of current health care providers, and increasing capacity by training new health care providers and addressing inequitable distribution of personnel. In terms of material resources, the focus should be on those interventions with the lowest cost-to-benefit ratio, such as antimicrobials and other Essential Medicines as recommended by the WHO, supplemental oxygen, and IV fluids. Adequate microbiology capacity is vital not only for the identification and targeted treatment of individual patients’ infection but also to inform local empiric therapeutic choices and pharmaceutical purchasing. One strategy to expand access to high-quality diagnostics is the development of regional reference laboratory networks [78, 79]. Additionally, as rapid diagnostic testing becomes more available and affordable, this may play a more important role in the initial management of sepsis. Indeed, this has already happened with the availability of inexpensive, rapid diagnostic test kits for malaria [80].

There is a great need for increased critical care capacity globally, and access to ICUs must be equitable. Building critical care capacity outside of the ICU is also important. This could be accomplished by training non-ICU medical staff, including nurses and paramedical assistants, in the principles of basic intensive care—careful monitoring and recording of vital signs, strict infection control practices, interdisciplinary daily rounds with the inclusion of patients and their families, and frequent reevaluation of patients at the bedside.

Increasing capacity of public health and acute health care delivery systems may introduce new ethical challenges. Ensuring that access to expanded acute health care resources is equitable, even when demand outstrips supply or patients have limited ability to pay, is a struggle even in the highest-resourced areas. Additional challenges potentially include greater survivorship burdens as patients are successfully treated for their sepsis but left with new chronic illness such as dialysis-dependent renal disease or cognitive or physical disability precluding return to work. While the difficult task of appropriately meeting needs of sepsis survivors is universal, these issues may be compounded in communities with high levels of poverty.

### Conduct inclusive research

**Fig. 3:**
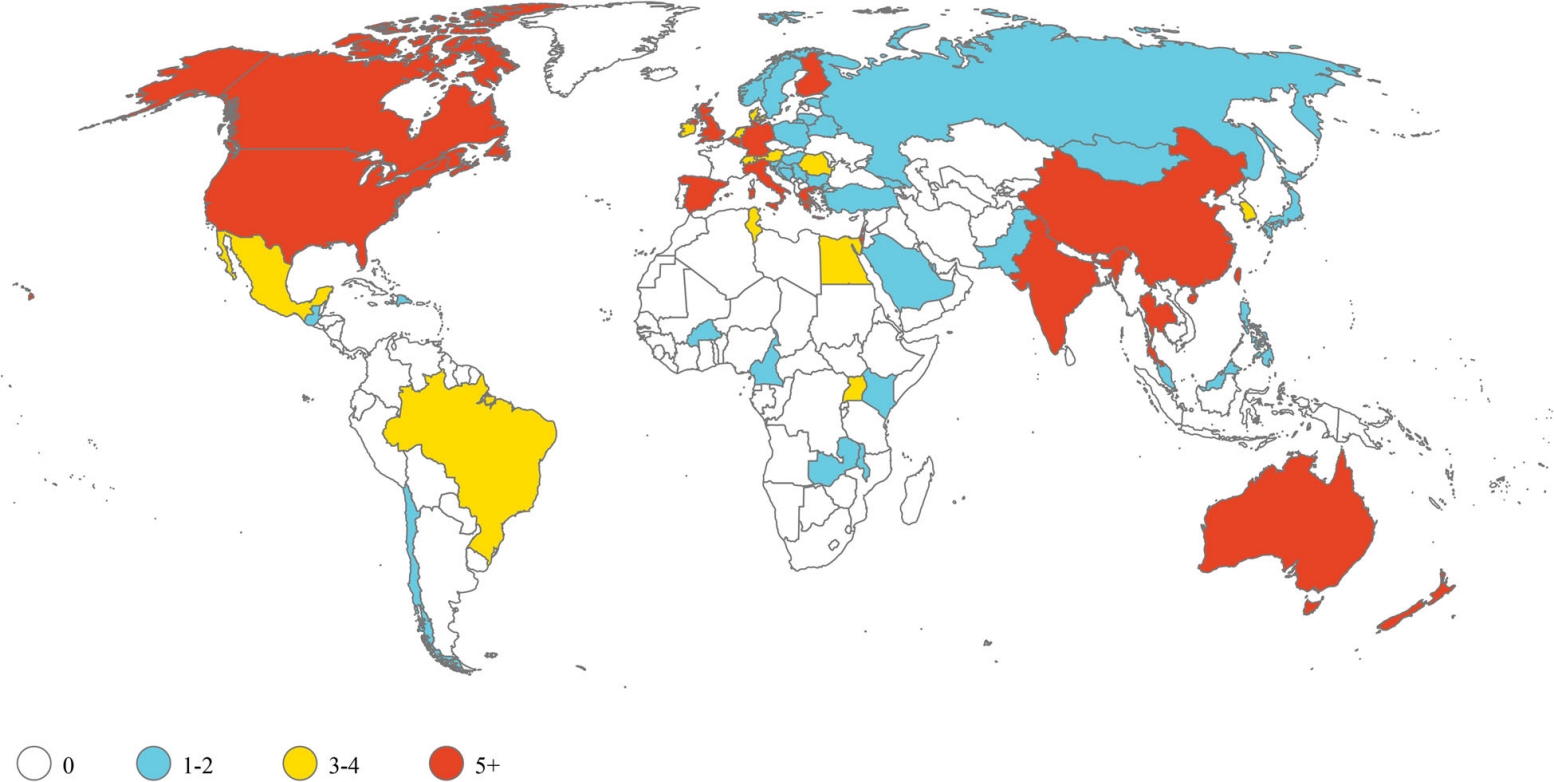
Sepsis trials are predominantly conducted in high-income countries. ClinicalTrials.gov and www.anzctr.org.au were searched on July 21, 2018 using search terminology “sepsis” in search terms and problem studied, study type “interventional”, and recruitment status “recruiting” or “enrolling by invitation”. Of 62 countries with any open interventional sepsis trial, 34 (55%) are classified as high-income economies by The World Bank

A roadmap for sepsis research has been developed [81] and can be further enhanced globally by increasing partnerships between the adult and pediatric sepsis research communities as well as integration of sepsis research with HIV, malaria, and other infectious disease research and clinical communities. The majority of sepsis trials registered on ClinicalTrials.gov (the US clinical trials registry, which is the largest in the world) and anzctr.org.au (the Australian New Zealand Clinical Trials Registry) are in HICs (Fig. 3). While there are some clinical studies that are not registered on these websites, this finding highlights the imbalances of some research resources. Private and public funding agencies have a scientific and moral obligation to ensure that research monies are appropriately directed to researchers from medically disadvantaged communities and those whose work focuses on correcting health inequities.

In light of the many unanswered questions about even the most basic treatment of patients with sepsis outside of high-resource settings (e.g., fluid resuscitation or the utility of point-of-care diagnostic tests), future interventional trials should include patients from low-resource settings. There is also a vital role for implementation science research within the fields of sepsis and critical care—how best to implement interventions proven to be effective, such as infection control practices, vaccines, or early antibiotics. Better understanding *how* to deliver lifesaving care in an effective, efficient, and affordable way is equally as important as understanding *what* interventions are safe and appropriate. Researchers in both high and low-resource settings should be prepared to make valuable data open access. This may necessitate the standardized inclusion of text describing open access in consent forms.

### Establish data-driven and context-specific management guidelines

Several groups have published guidelines for the management of sepsis in low-resource settings [82, 83]. Although laudable and an important step forward, these guidelines are largely nonevidence-based for low-resource environments and reflect care in high-resource settings, which may not be available or appropriate. Additionally, many LMICs heavily utilize WHO guidelines for the integrated management of childhood or adult and adolescent illness (IMCI and IMAI). The IMAI guidelines for district clinicians include recommendations on the identification and management of patients with septic shock and respiratory failure without shock [84]. However, the guidelines do not specifically address sepsis management in the absence of shock or respiratory failure, and are largely organized around individual infectious syndromes. Given this approach, many clinicians in areas utilizing the WHO guidelines may not specifically document the presence of sepsis when present, instead focusing on syndromically defined diseases.

The core of any recommendations must focus on the implementation of interventions that are high yield, cost-effective, and readily available in low-resource settings. Additionally, these recommendations must be developed in an iterative process, continually undergoing rigorous evaluation for effectiveness in low-resource settings and being updated as appropriate. Recommendations for or against specific interventions in variably resourced environments should be provided, and management guidelines should include disease-specific recommendations (e.g., for dengue). It is critical to partner with a diverse spectrum of health care providers, public health professionals, and researchers to accomplish this objective, educate front-line staff, and implement recommendations.

### Promote creative interventions

Both simple and inventive strategies can help to save many lives from sepsis in low-resource settings and should be encouraged. The absence of ICUs or advanced technologies such as mechanical ventilators or invasive monitoring does not preclude the provision of many important components of sepsis care, such as source control and early appropriate antimicrobials [85, 86]. Implementation of these components can be further augmented by creative approaches to sepsis management such as timely pre-referral rectal administration of antimicrobials in basic health centers or use of intraosseous access for parenteral therapies [87].

### Advocacy

The WHO initiative on maternal and neonatal sepsis and the Global Sepsis Alliance are examples of international initiatives tackling the global burden of sepsis. In addition to increasing awareness and promoting research and education, broader engagement with policymakers and government is required to identify opportunities to improve data collection, care processes, and outcomes.

## Conclusions

While the global burden of sepsis is vast, we offer specific steps necessary to reduce its human and economic toll in low-resource settings. Most importantly, sepsis must be viewed as a major international public health problem. Accordingly, efforts to address it must focus fundamentally on economic inequity, social determinants of health, and public health infrastructure.

## Abbreviations

AMR: Antimicrobial resistance
HIC: High-income country
HIV: Human Immunodeficiency Virus
ICD: International Classification of Diseases
ICU: Intensive care unit
IHME: Institute for Health Metrics and Evaluation
IV: Intravenous
LMIC: Low and middle-income country
RCT: Randomized clinical trial
SIRS: Systemic inflammatory response syndrome
WHO: World Health Organization

## References

[1] Fleischmann C, Scherag A, Adhikari NKJ, Hartog CS, Tsaganos T, Schlattmann P (2016). Assessment of global incidence and mortality of hospital-treated sepsis—current estimates and limitations. Am J Respir Crit Care Med.

[2] Gaieski DF, Edwards JM, Kallan MJ, Carr BG (2013). Benchmarking the incidence and mortality of severe sepsis in the United States. Crit Care Med.

[3] Rhee C, Dantes R, Epstein L, Murphy DJ, Seymour CW, Iwashyna TJ (2017). Incidence and trends of sepsis in US hospitals using clinical vs claims data, 2009-2014. JAMA.

[4] Wilhelms SB, Huss FR, Granath G, Sjöberg F (2010). Assessment of incidence of severe sepsis in Sweden using different ways of abstracting International Classification of Diseases codes: difficulties with methods and interpretation of results. Crit Care Med.

[5] Jawad I, Lukšić I, Rafnsson SB (2012). Assessing available information on the burden of sepsis: global estimates of incidence, prevalence and mortality. J Glob Health.

[6] Kaukonen K-M, Bailey M, Suzuki S, Pilcher D, Bellomo R (2014). Mortality related to severe sepsis and septic shock among critically ill patients in Australia and New Zealand, 2000-2012. JAMA.

[7] Kumar A, Roberts D, Wood KE, Light B, Parrillo JE, Sharma S (2006). Duration of hypotension before initiation of effective antimicrobial therapy is the critical determinant of survival in human septic shock. Crit Care Med.

[8] Bone RC, Balk RA, Cerra FB, Dellinger RP, Fein AM, Knaus WA (1992). Definitions for sepsis and organ failure and guidelines for the use of innovative therapies in sepsis. Chest.

[9] Singer M, Deutschman CS, Seymour CW, Shankar-Hari M, Annane D, Bauer M (2016). The third international consensus definitions for sepsis and septic shock (Sepsis-3). JAMA.

[10] Seymour CW, Opal SM, Rubenfeld GD, van der Poll T, Vincent J, Angus DC (2016). Assessment of clinical criteria for sepsis for the third international consensus definitions for sepsis and septic shock (Sepsis-3). JAMA.

[11] Rudd Kristina E., Seymour Christopher W., Aluisio Adam R., Augustin Marc E., Bagenda Danstan S., Beane Abi, Byiringiro Jean Claude, Chang Chung-Chou H., Colas L. Nathalie, Day Nicholas P. J., De Silva A. Pubudu, Dondorp Arjen M., Dünser Martin W., Faiz M. Abul, Grant Donald S., Haniffa Rashan, Van Hao Nguyen, Kennedy Jason N., Levine Adam C., Limmathurotsakul Direk, Mohanty Sanjib, Nosten François, Papali Alfred, Patterson Andrew J., Schieffelin John S., Shaffer Jeffrey G., Thuy Duong Bich, Thwaites C. Louise, Urayeneza Olivier, White Nicholas J., West T. Eoin, Angus Derek C. (2018). Association of the Quick Sequential (Sepsis-Related) Organ Failure Assessment (qSOFA) Score With Excess Hospital Mortality in Adults With Suspected Infection in Low- and Middle-Income Countries. JAMA.

[12] Goldstein B, Giroir B, Randolph A (2005). International pediatric sepsis consensus conference: definitions for sepsis and organ dysfunction in pediatrics. Pediatr Crit Care Med.

[13] Wiens MO, Kumbakumba E, Kissoon N, Ansermino JM, Ndamira A, Larson CP (2012). Pediatric sepsis in the developing world: challenges in defining sepsis and issues in post-discharge mortality. Clin Epidemiol.

[14] Weiss SL, Fitzgerald JC, Maffei FA, Kane JM, Rodriguez-Nunez A, Hsing DD (2015). Discordant identification of pediatric severe sepsis by research and clinical definitions in the SPROUT international point prevalence study. Crit Care.

[15] Kissoon N, Uyeki TM (2015). Sepsis and the global burden of disease in children. JAMA Pediatr.

[16] GBD 2013 Mortality and Causes of Death Collaborators (2015). Global, regional, and national age–sex specific all-cause and cause-specific mortality for 240 causes of death, 1990–2013: a systematic analysis for the Global Burden of Disease Study 2013. Lancet.

[17] World Health Organization (2015). World Health Statistics 2015.

[18] Seventieth World Health Assembly. Improving the prevention, diagnosis and clinical management of sepsis. Geneva: World Health Organization; 2017.

[19] Gavidia R, Fuentes SL, Vasquez R, Bonilla M, Ethier MC, Diorio C (2012). Low socioeconomic status is associated with prolonged times to assessment and treatment, sepsis and infectious death in pediatric fever in El Salvador. PLoS One.

[20] von Mollendorf C, Cohen C, de Gouveia L, Naidoo N, Meiring S, Quan V (2015). Risk factors for invasive pneumococcal disease among children less than 5 years of age in a high HIV-prevalence setting, South Africa, 2010 to 2012. Pediatr Infect Dis J.

[21] Jackson S, Mathews KH, Pulanic D, Falconer R, Rudan I, Campbell H (2013). Risk factors for severe acute lower respiratory infections in children—a systematic review and meta-analysis. Croat Med J.

[22] Upadhyay AK, Singh A, Kumar K, Singh A (2015). Impact of indoor air pollution from the use of solid fuels on the incidence of life threatening respiratory illnesses in children in India. BMC Public Health.

[23] The World Bank. DataBank. http://databank.worldbank.org/data/reports.aspx?source=health-nutrition-and-population-statistics. Accessed 22 Mar 2018.

[24] Manongi R, Mtei F, Mtove G, Nadjm B, Muro F, Alegana V (2014). Inpatient child mortality by travel time to hospital in a rural area of Tanzania. Trop Med Int Heal.

[25] Global Health Workforce Alliance, World Health Organization (2013). A universal truth: no health without a workforce.

[26] Peltola L, Goddia C, Namboya F, Brunkhorst F, Pollach G (2015). Sepsis—knowledge of non-physician personnel in Africa: a cross-sectional study in Malawian district hospitals. Med Klin Intensivmed Notfmed.

[27] Guo A, Bowling JM, Bartram J, Kayser G (2017). Water, sanitation, and hygiene in rural health-care facilities: a cross-sectional study in Ethiopia, Kenya, Mozambique, Rwanda, Uganda, and Zambia. Am J Trop Med Hyg..

[28] Alkire BC, Raykar NP, Shrime MG, Weiser TG, Bickler SW, Rose JA (2015). Global access to surgical care: a modelling study. Lancet Glob Heal.

[29] Uribe-Leitz T, Esquivel MM, Molina G, Lipsitz SR, Verguet S, Rose J (2015). Projections for achieving the lancet commission recommended surgical rate of 5000 operations per 100,000 population by region-specific surgical rate estimates. World J Surg.

[30] Okoye MT, Ameh EA, Kushner AL, Nwomeh BC (2015). A pilot survey of pediatric surgical capacity in West Africa. World J Surg.

[31] Murthy S, Leligdowicz A, Adhikari NK (2015). Intensive care unit capacity in low-income countries: a systematic review. PLoS One.

[32] Kissoon N, Burns J (2014). Who should get pediatric intensive care when not all can? A call for international guidelines on allocation of pediatric intensive care resources. Pediatr Crit Care Med.

[33] Baelani I, Jochberger S, Laimer T, Rex C, Baker T, Wilson IH (2012). Identifying resource needs for sepsis care and guideline implementation in the democratic republic of the Congo: a cluster survey of 66 hospitals in four eastern provinces. Middle East J Anesthesiol.

[34] Belle J, Cohen H, Shindo N, Lim M, Velazquez-Berumen A, Ndihokubwayo JB (2010). Influenza preparedness in low-resource settings: a look at oxygen delivery in 12 African countries. J Infect Dev Ctries.

[35] Pollach G, Namboya F (2013). Preventing intensive care admissions for sepsis in tropical Africa (PICASTA): an extension of the international pediatric global sepsis initiative: an African perspective. Pediatr Crit Care Med.

[36] Rivers E, Nguyen B, Havstad S, Ressler J, Muzzin A, Knoblich B (2001). Early goal-directed therapy in the treatment of severe sepsis and septic shock. N Engl J Med.

[37] Rhodes A, Evans LE, Alhazzani W, Levy MM, Antonelli M, Ferrer R (2017). Surviving Sepsis Campaign: International Guidelines for Management of Sepsis and Septic Shock: 2016. Crit Care Med.

[38] Andrews B, Muchemwa L, Kelly P, Lakhi S, Heimburger DC, Bernard GR (2014). Simplified severe sepsis protocol: a randomized controlled trial of modified early goal-directed therapy in Zambia. Crit Care Med.

[39] Pepper DJ, Jaswal D, Sun J, Welsh J, Natanson C, Eichacker PQ (2018). Evidence underpinning the Centers for Medicare & Medicaid Services’ Severe Sepsis and Septic Shock Management Bundle (SEP-1): a systematic review. Ann Intern Med.

[40] Rhodes A, Evans LE, Alhazzani W, Levy MM, Antonelli M, Ferrer R, et al. Surviving Sepsis Campaign: International Guidelines for Management of Sepsis and Septic Shock: 2016. Intensive Care Med. 2017;43:304-77. 10.1007/s00134-017-4683-6 28101605

[41] Orozco JD, Greenberg LA, Desai IK, Anglade F, Ruhangaza D, Johnson M (2018). Building laboratory capacity to strengthen health systems: the partners in health experience. Clin Lab Med.

[42] Kelesidis T, Kelesidis I, Rafailidis PI, Falagas ME (2007). Counterfeit or substandard antimicrobial drugs: a review of the scientific evidence. J Antimicrob Chemother.

[43] Newton PN, Green MD, Fernandez FM, Day NPJ, White NJ (2006). Counterfeit anti-infective drugs. Lancet Infect Dis.

[44] Akande TM, Aderibigbe SA (2007). Influence of drug promotion on prescribing habits of doctors in a teaching hospital. Afr J Med Med Sci.

[45] Kamal S, Holmberg C, Russell J, Bochenek T, Tobiasz-Adamczyk B, Fischer C (2015). Perceptions and attitudes of Egyptian health professionals and policy-makers towards pharmaceutical sales representatives and other promotional activities. PLoS One.

[46] Ntirenganya C, Manzi O, Muvunyi CM, Ogbuagu O (2015). High prevalence of antimicrobial resistance among common bacterial isolates in a tertiary healthcare facility in Rwanda. Am J Trop Med Hyg..

[47] Bataar O, Khuderchuluun C, Lundeg G, Chimeddorj S, Brunauer A, Gradwohl-Matis I (2013). Rate and pattern of antibiotic resistance in microbiological cultures of sepsis patients in a low-middle-income country’s ICU. Middle East J Anesthesiol.

[48] Moehario LH, Tjoa E, Kiranasari A, Ningsih I, Rosana Y, Karuniawati A (2009). Trends in antimicrobial susceptibility of gram-negative bacteria isolated from blood in Jakarta from 2002 to 2008. J Infect Dev Ctries..

[49] Review on Antimicrobial Resistance (2014). Antimicrobial resistance: tackling a crisis for the health and wealth of nations.

[50] Tadesse BT, Ashley EA, Ongarello S, Havumaki J, Wijegoonewardena M, González IJ (2017). Antimicrobial resistance in Africa: a systematic review. BMC Infect Dis.

[51] Hay SI, Rao PC, Dolecek C, Day NPJ, Stergachis A, Lopez AD (2018). Measuring and mapping the global burden of antimicrobial resistance. BMC Med.

[52] World Health Organization (2014). Antimicrobial Resistance: Global Report on Surveillance 2014.

[53] Zusman O, Paul M, Farbman L, Daitch V, Akayzen Y, Witberg G (2015). Venous thromboembolism prophylaxis with anticoagulation in septic patients: a prospective cohort study. Q J Med.

[54] Bamgboye EL (2016). The challenges of ESRD care in developing economies: sub-Saharan African opportunities for significant improvement. Clin Nephrol.

[55] Alobaidi R, Basu RK, Goldstein SL, Magshaw SM (2015). Sepsis-associated acute kidney injury. Semin Nephrol.

[56] Pittet D, Allegranzi B, Storr J, Nejad SB, Dziekan G, Leotsakos A (2008). Infection control as a major World Health Organization priority for developing countries. J Hosp Infect.

[57] Weiss SL, Fitzgerald JC, Pappachan J, Wheeler D, Jaramillo-Bustamante JC, Salloo A (2015). Global epidemiology of pediatric severe sepsis: the sepsis prevalence, outcomes, and therapies study. Am J Respir Crit Care Med.

[58] Wiens MO, Pawluk S, Kissoon N, Kumbakumba E, Ansermino JM, Singer J (2013). Pediatric post-discharge mortality in resource poor countries: a systematic review. PLoS One.

[59] Wiens MO, Kumbakumba E, Larson CP, Ansermino JM, Singer J, Kissoon N (2015). Postdischarge mortality in children with acute infectious diseases: derivation of postdischarge mortality prediction models. BMJ Open.

[60] Ranjit S, Aram G, Kissoon N, Ali MK, Natraj R, Shresti S (2014). Multimodal monitoring for hemodynamic categorization and management of pediatric septic shock: a pilot observational study. Pediatr Crit Care Med.

[61] Tulloch LG, Chan JD, Carlbom DJ, Kelly MJ, Dellit TH, Lynch JB (2017). Epidemiology and microbiology of sepsis syndromes in a university-affiliated urban teaching hospital and Level-1 trauma and burn center. J Intensive Care Med.

[62] Southeast Asia Infectious Disease Clinical Research Network (2017). Causes and outcomes of sepsis in Southeast Asia: a multinational multicentre cross-sectional study. Lancet Glob Heal..

[63] Heffner AC, Horton JM, Marchick MR, Jones AE (2010). Etiology of illness in patients with severe sepsis admitted to the hospital from the emergency department. Clin Infect Dis.

[64] Kanoksil M, Jatapai A, Peacock SJ, Limmathurotsakul D (2013). Epidemiology, microbiology and mortality associated with community-acquired bacteremia in northeast Thailand: a multicenter surveillance study. PLoS One.

[65] Feasey NA, Dougan G, Kingsley RA, Heyderman RS, Gordon MA (2012). Invasive non-typhoidal salmonella disease: an emerging and neglected tropical disease in Africa. Lancet.

[66] Reddy EA, Shaw AV, Crump JA (2010). Community-acquired bloodstream infections in Africa: a systematic review and meta-analysis. Lancet Infect Dis.

[67] Limmathurotsakul D, Wongratanacheewin S, Teerawattanasook N, Wongsuvan G, Chaisuksant S, Chetchotisakd P (2010). Increasing incidence of human melioidosis in northeast Thailand. Am J Trop Med Hyg..

[68] Jacob ST, Moore CC, Banura P, Pinkerton R, Meya D, Opendi P (2009). Severe sepsis in two Ugandan hospitals: a prospective observational study of management and outcomes in a predominantly HIV-1 infected population. PLoS One.

[69] Bell M, Archibald LK, Nwanyanwu O, Dobbie H, Tokars J, Kazembe PN (2001). Seasonal variation in the etiology of bloodstream infections in a febrile inpatient population in a developing country. Int J Infect Dis.

[70] Mayxay M, Castonguay-Vanier J, Chansamouth V, Dubot-Pérès A, Paris DH, Phetsouvanh R (2013). Causes of non-malarial fever in Laos: a prospective study. Lancet Glob Heal..

[71] Moore CC, Jacob ST, Pinkerton R, Banura P, Meya DB, Reynolds SJ (2009). Treatment of severe sepsis with artemether-lumefantrine is associated with decreased mortality in Ugandan patients without malaria. Am J Trop Med Hyg.

[72] Hanson J, Anstey NM, Bihari D, White NJ, Day NP, Dondorp AM (2014). The fluid management of adults with severe malaria. Crit Care.

[73] Maude RJ, Hoque G, Hasan MU, Sayeed A, Akter S, Samad R (2011). Timing of enteral feeding in cerebral malaria in resource-poor settings: a randomized trial. PLoS One.

[74] Maitland K, Kiguli S, Opoka RO, Engoru C, Olupot-Olupot P, Akech SO (2011). Mortality after fluid bolus in African children with severe infection. N Engl J Med.

[75] Andrews B, Semler MW, Muchemwa L, Kelly P, Lakhi S, Heimburger DC (2017). Effect of an early resuscitation protocol on in-hospital mortality among adults with sepsis and hypotension: a randomized clinical trial. JAMA.

[76] Kissoon N, Carcillo JA, Espinosa V, Argent A, Devictor D, Madden M (2011). World Federation of Pediatric Intensive Care and Critical Care Societies: Global Sepsis Initiative. Pediatr Crit Care Med.

[77] Guerra WF, Mayfield TR, Meyers MS, Clouatre AE, Riccio JC (2013). Early detection and treatment of patients with severe sepsis by prehospital personnel. J Emerg Med.

[78] Schneidman M, Matu M, Nkengasong J, Githui W, Kalyesubula-Kibuuka S, Silva KA (2018). Building cross-country networks for laboratory capacity and improvement. Clin Lab Med.

[79] Semret M, Ndao M, Jacobs J, Yansouni CP (2018). Point-of-care and point-of-“can”: leveraging reference-laboratory capacity for integrated diagnosis of fever syndromes in the tropics. Clin Microbiol Infect.

[80] Dinko B, Amakpa E, Kweku M, Amoah P, Tampuori J, Adjuik M (2018). Plasmodium falciparum malaria cases detected for prompt treatment by rapid diagnostic tests in the Ho Teaching Hospital of the Volta Region of Ghana. Parasite Epidemiol Control.

[81] Cohen J, Vincent JL, Adhikari NKJ, Machado FR, Angus DC, Calandra T (2015). Sepsis: a roadmap for future research. Lancet Infect Dis.

[82] Thwaites CL, Lundeg G, Dondorp AM (2016). Recommendations for infection management in patients with sepsis and septic shock in resource-limited settings. Intensive Care Med.

[83] Serpa Neto A, Schultz MJ, Festic E (2016). Ventilatory support of patients with sepsis or septic shock in resource-limited settings. Intensive Care Med.

[84] Jacob ST, Lim M, Banura P, Bhagwanjee S, Bion J, Cheng AC (2013). Integrating sepsis management recommendations into clinical care guidelines for district hospitals in resource-limited settings: the necessity to augment new guidelines with future research. BMC Med.

[85] Baker T, Schell CO, Lugazia E, Blixt J, Mulungu M, Castegren M (2015). Vital signs directed therapy: improving care in an intensive care unit in a low-income country. PLoS One.

[86] Chisti MJ, Salam MA, Smith JH, Ahmed T, Pietroni MAC, Shahunja KM (2015). Bubble continuous positive airway pressure for children with severe pneumonia and hypoxaemia in Bangladesh: an open, randomised controlled trial. Lancet.

[87] Gomes MF, Faiz MA, Gyapong JO, Warsame M, Agbenyega T, Babiker A (2009). Pre-referral rectal artesunate to prevent death and disability in severe malaria: a placebo-controlled trial. Lancet.

